# Impact of small area-level deprivation on survival outcomes in patients with Cholangiocarcinoma: a population-based multilevel survival analysis

**DOI:** 10.3389/fpubh.2026.1771770

**Published:** 2026-04-30

**Authors:** Jonghyun Lee, Ahreum Kim, Seunghye Jang, Changhoon Kim, Dong Uk Kim, Sung Yong Han, Seung Hun Lee, Yu Hyeon Yi, Heeseung Park, Kiyoun Yi, Seung Min Hong, Hyun Joo Lee

**Affiliations:** 1 Department of Internal Medicine, School of Medicine, Pusan National University, Busan, Republic of Korea; 2 Division of Gastroenterology, Department of Internal Medicine and Biomedical Research Institute, Busan National University Hospital, Busan, Republic of Korea; 3 Office of Public Healthcare Service, Pusan National University Hospital, Busan, Republic of Korea; 4 Biomedical Research Institute, Pusan National University Hospital, Busan, Republic of Korea; 5 Department of Preventive Medicine, School of Medicine, Pusan National University, Busan, Republic of Korea; 6 Gumi Medical Center, Department of Internal Medicine, CHA University, Gumi, Republic of Korea; 7 Department of Family Medicine, School of Medicine, Pusan National University, Busan, Republic of Korea; 8 Department of Family Medicine and Biomedical Research Institute, Pusan National University Hospital, Busan, Republic of Korea; 9 Department of Surgery, School of Medicine, Pusan National University, Busan, Republic of Korea; 10 Department of Surgery and Biomedical Research Institute, Pusan National University Hospital, Busan, Republic of Korea; 11 Department of Obstetrics and Gynecology, School of Medicine, Pusan National University, Busan, Republic of Korea

**Keywords:** aging population, Cholangiocarcinoma, deprivation index, socioeconomic accessibility, survival analysis

## Abstract

**Background:**

Cholangiocarcinoma (CCA) carries a dismal prognosis. Beyond clinical determinants, socioeconomic deprivation may also influence outcomes. Busan, a rapidly aging metropolitan area in Korea, provides a critical setting for evaluating how area-level deprivation interacts with individual risk factors for CCA survival. However, evidence regarding the effect of area-level deprivation on CCA survival remains scarce, particularly within the Korean population. We aimed to evaluate the association between the deprivation index (DI) and individual clinical factors and provide a deeper understanding of the variability in the survival rates of patients with CCA in Busan.

**Methods:**

We analyzed data from 12,950 patients diagnosed with CCA between 2003 and 2020 using the Busan Regional Cancer Registry linked with national death records. Individual-level variables included age, sex, stage, and year of diagnosis, while area-level deprivation was quantified using the census-derived DI. Multilevel survival analyses were performed using a frailty Cox proportional hazards model with a random intercept for region to estimate hazard ratios (HRs) for both individual- and regional-level predictors.

**Results:**

The median survival period was 42 weeks. Survival declined with age (46.5% vs. 21.3% in patients aged 0–44 years and ≥75 years, respectively, *p* < 0.001) and was significantly lower in advanced-stage disease (47.7% localized vs. 8.07% distant, *p* < 0.001). Men had marginally higher survival rates than women (33.8% vs. 32.1%, p < 0.001). Deprivation strongly influenced prognosis, as patients in the most deprived areas had a 30.1% survival rate compared with 36.3% in the least deprived areas (p < 0.001). In fully adjusted models, age ≥75 years (HR 2.68), regional (HR 1.40) and distant stages (HR 3.77), and higher DI (HR 1.06 per unit) independently predicted poorer survival. Incorporating DI into the models slightly reduced the between-region variance (*σ*) and median hazard ratio (MHR), indicating that area-level deprivation partially explains regional differences in survival outcomes.

**Conclusion:**

CCA survival in Busan is influenced by both individual vulnerability and area-level deprivation. Older age, advanced stage, and higher deprivation were identified as independent poor prognostic factors for CCA survival. In super-aged urban societies, integrating social determinants into clinical care is essential to reduce inequities and improve outcomes.

## Highlights

1. Provides population-based multilevel survival analysis of Cholangiocarcinoma in Korea linking small-area deprivation with outcomes.
2. Demonstrates that neighborhood deprivation independently predicts poorer survival beyond age, sex, stage, and year of diagnosis.
3. Quantifies how deprivation explains regional survival differences using measures such as the deprivation index and median hazard ratio.
4. Highlights that older adults in deprived urban neighborhoods face the greatest survival disadvantage, informing equity-focused cancer control in super-aged societies.

## Background

Cholangiocarcinoma (CCA) is a highly aggressive malignancy of the biliary epithelium and represents a major clinical challenge owing to its late presentation and poor prognosis (1, 2). CCA predominantly occurs in older populations, with survival varying substantially by stage at diagnosis (3). Globally, the incidence of CCA shows marked geographical variation, with a relatively high prevalence in East and Southeast Asia. In Korea, the burden of CCA has remained substantial despite advances in diagnostics and therapy, making it a critical focus for regional cancer control (4, 5).

Survival outcomes in CCA are influenced not only by established clinical factors such as age, sex, and tumor stage at diagnosis, but also by broader socioeconomic determinants of health. Socioeconomic deprivation, which reflects cumulative disadvantages in housing, education, occupation, and living environment, has emerged as an important contextual factor in cancer epidemiology (6, 7). Patients from deprived neighborhoods often experience delayed diagnosis, limited access to specialized care, and worse survival outcomes across various malignancies (8–10). Socioeconomic disparities may influence cancer survival through multiple mechanisms, including delayed diagnosis, reduced access to specialized hepatobiliary centers, and differences in treatment allocation. In Cholangiocarcinoma, previous studies using large-scale databases have demonstrated that patients from socioeconomically disadvantaged areas are less likely to receive surgical resection or advanced oncologic therapies, which may contribute to poorer survival outcomes (11–13). However, evidence regarding the effect of area-level deprivation on CCA survival remains scarce, particularly within the Korean population.

Busan, Korea’s second-largest metropolitan city, presents a unique context for this study. The city is undergoing a rapid demographic transition into a super-aged society, with one of the fastest-growing older populations in the country. This demographic shift is expected to amplify the impact of area-level deprivation on cancer outcomes, as older individuals are particularly vulnerable to socioeconomic disadvantages and barriers in healthcare access. Consequently, the interaction between age distribution and regional deprivation in Busan provides a compelling framework for examining the health inequities in CCA survival.

Therefore, we aimed to assess the effect of small-area deprivation on survival outcomes among patients with CCA in Busan, using population-based data from the Busan Regional Cancer Registry (BRCR). Through multilevel survival analysis, we examined how neighborhood-level deprivation, alongside individual-level factors such as age, sex, and cancer stage, contributes to prognostic variations in patients with CCA. We hypothesized that higher area-level deprivation independently worsens survival outcomes in patients with CCA, even after adjusting for individual-level clinical factors. These findings may provide evidence to inform region-specific cancer control strategies and health policy interventions aimed at reducing socioeconomic disparities in survival outcomes.

## Methods

### Data source

Data for this study were obtained from the BRCR, a subset of the Korea Central Cancer Registry, which collects nationwide cancer incidence data (14). The completeness of cancer registration is estimated to be 97.8% (15). We analyzed CCA data from the Busan population (*N* = 12,950) between 2003 and 2020. To confirm the dates and causes of death, BRCR data were linked with national death records. Follow-up extended from the date of diagnosis to December 31, 2022. Patients with missing key variables (age, sex, stage, or survival time) or incomplete linkage to mortality data were excluded from the analysis.

### Outcome variable

The outcome variable was CCA-specific survival, defined as the time from diagnosis to death or censoring. Primary diagnoses of CCA within the study cohort were confirmed using BRCR records, restricted to cases classified as CCA under the 10th revision of the International Classification of Diseases (ICD-10). Mortality due to CCA (ICD-10: C23–C24, C221) was verified using the national cause of death registry for the same period.

### Explanatory variables

At the individual level, independent variables included age group (0–44, 45–64, 65–74, ≥75 years), sex, cancer stage (localized, regional, distant), and year of diagnosis. Age was categorized into clinically meaningful groups to reflect differences in treatment eligibility and physiological vulnerability across life stages. At the regional level, individuals were grouped into 205 administrative neighborhoods based on their residence. These neighborhoods represent the smallest administrative units in which community services are provided through welfare centers. The deprivation index (DI) is employed as a regional-level explanatory variable. DI was calculated following previously described methods (16, 17), using data from the 2015 Population and Housing Census. It incorporates factors such as housing environment, education, social class, older population, single-person households, car-free households, non-apartment housing, female-headed households, and divorced or separated households. A higher DI score indicates a higher level of deprivation. For analysis, DI was modeled both as a continuous variable (per unit increase) and as categorical quintiles (1–19%, 20–39%, 40–59%, 60–79%, and 80–100%), with higher categories representing greater deprivation.

### Statistical analysis

**Figure 1:**
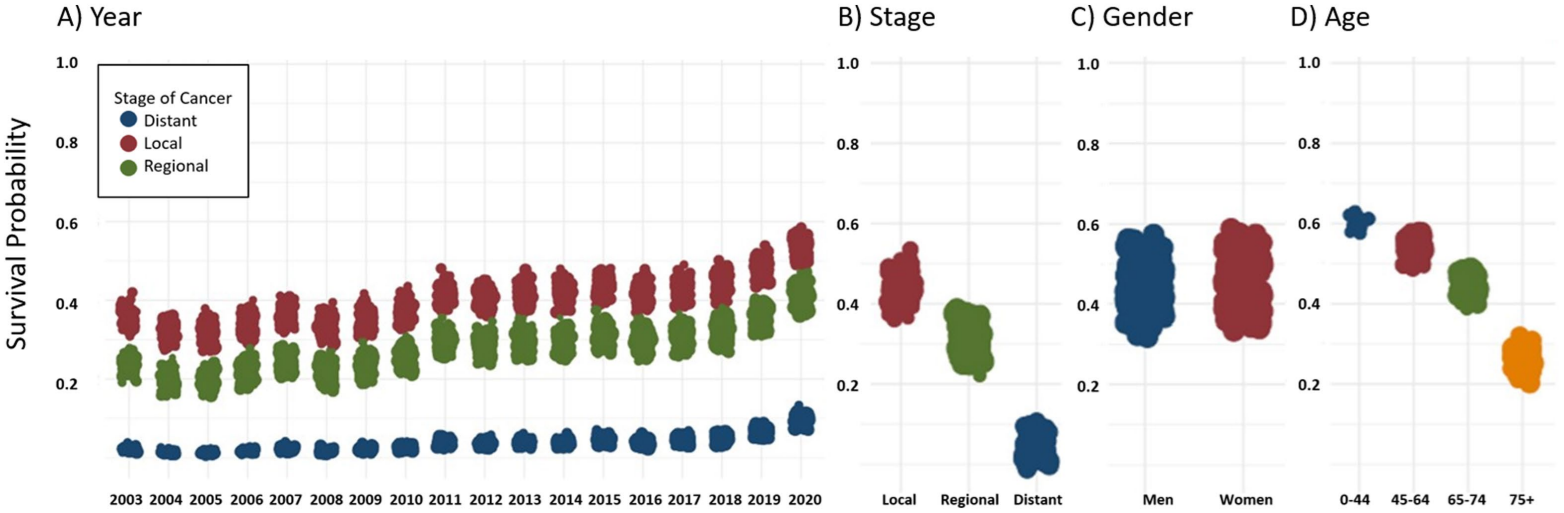
Adjusted marginal survival probabilities derived from the multilevel frailty Cox model. Estimates reflect model-based predictions rather than unadjusted Kaplan–Meier survival.

**Figure 2:**
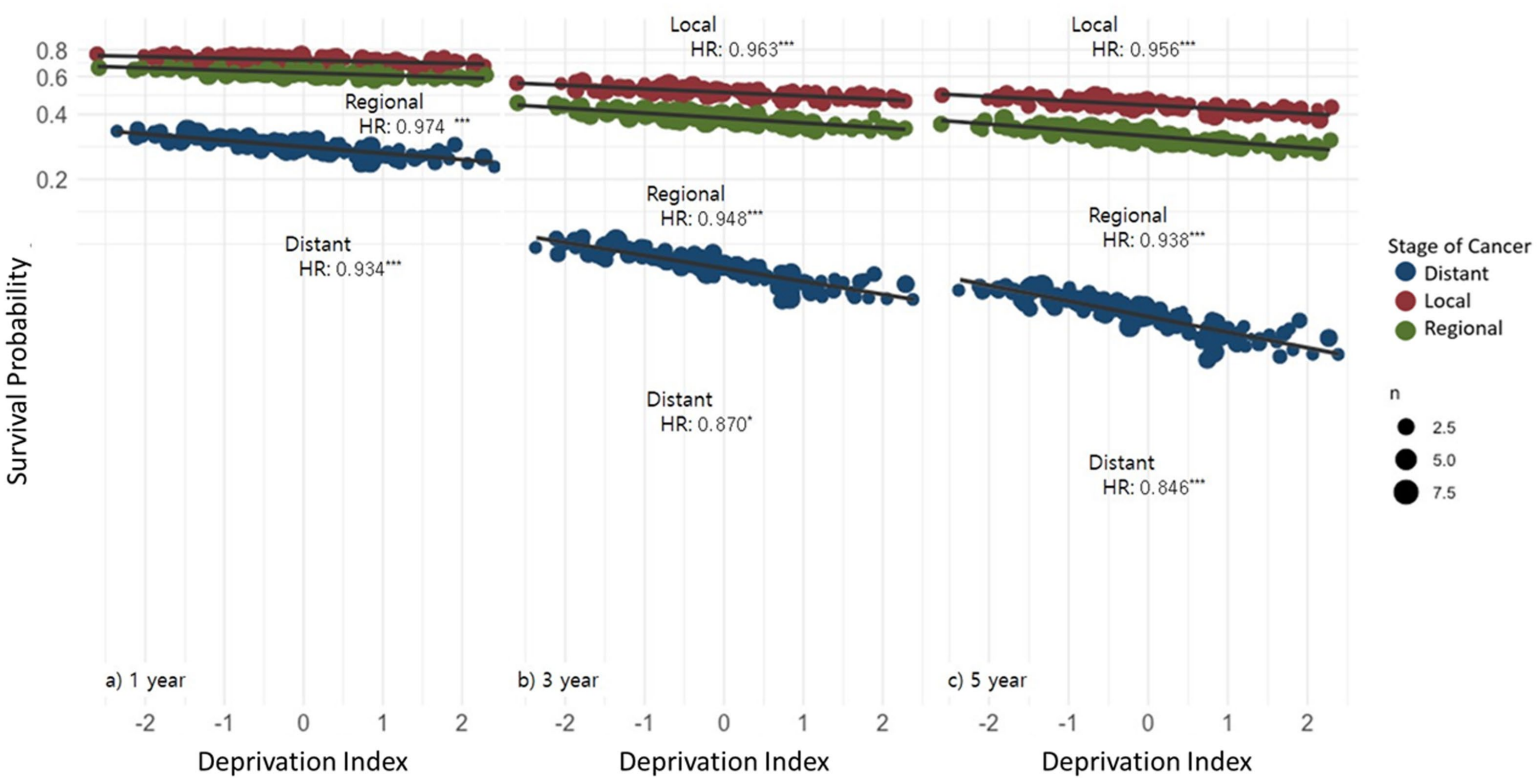
Association between stage at diagnosis and deprivation level with 1-, 3-, and 5-year area-level survival probability.

The Kaplan–Meier method was used to estimate median survival times across factors influencing survival, and group comparisons were conducted using the log-rank test. Differences in categorical variables were assessed using the chi-square test. Temporal trends in cancer diagnoses were evaluated descriptively. To account for clustering of individuals within regions, a multilevel survival analysis was conducted using a frailty Cox proportional hazards model with a random intercept for region. This approach enabled the simultaneous evaluation of individual-level factors and regional-level effects on survival outcomes while accounting for unobserved heterogeneity between regions. Statistical significance was defined as a two-tailed *p*-value < 0.05. To evaluate the relationship between individual-level risk factors and regional characteristics, the model was progressively refined by adding individual and regional variables, moving from a simple to a more complex structure. An initial “empty” model with random intercepts was estimated to assess regional differences. Subsequently, individual characteristics were incorporated to determine the extent to which these differences were explained by individual composition. Hazard ratios (HRs) were calculated to represent the relative risk for each category compared with the reference category (HR = 1 indicates the reference group; HR > 1 indicates increased risk). The frailty model was chosen to account for clustering at the regional level and between-region variability, which is particularly relevant in population-based studies with hierarchical data structures and may not be fully addressed by a conventional Cox proportional hazards model. Finally, the regional DI was incorporated to assess the explanatory power of regional-level variance and to evaluate whether regional differences were adjusted for regional characteristics (18, 19). To directly compare the contribution of individual-level risk factors and regional-level variation, the median hazard ratio (MHR) was reported as a measure of group heterogeneity (clustering effect). Empirical Bayesian estimation was applied to derive regional-level estimates from the analysis model. Results are presented in Figure 1, showing the marginal effect estimations for each factor. To highlight the gradient of the DI, survival rates on the Y-axis were log-transformed, and the HRs are shown in Figure 2.

### Ethical considerations

This study was conducted in accordance with the ethical principles of the Declaration of Helsinki (2013 revision) and was reviewed by the Institutional Review Board (IRB) of Pusan National University Hospital. As the study utilized existing data from the BRCR without direct patient involvement and contained no personal identifiers, it was granted exemption approval (IRB ID: H-1412-012-024).

## Results

A total of 12,950 patients with CCA were identified from the Busan Cancer Registry from 2003 to 2020. Table 1 presents the survival probabilities stratified by patient characteristics. Most patients were aged 65–74 years (35.3%, *n* = 4,576), male (52.8%, *n* = 6,839), and diagnosed at a regional stage (41.6%, *n* = 5,383). CCA diagnoses increased over the study period. Distribution across deprivation levels was relatively even, with 20.7% in the least deprived group and 19.6% in the most deprived group (*p* = 0.065).

**Table 1:** Survival probabilities of Cholangiocarcinoma according to study characteristics.

		Cancer patients		Median survival	
		Number (%)	*p*-value	Probability (%)	*p*-value
Age group	0–44	231 (1.8%)	<0.001	46.5 (39.9, 54.2)	<0.001
	45–64	4,060 (31.4%)		41.3 (39.7, 43.0)	
	65–74	4,576 (35.3%)		34.9 (33.4, 36.4)	
	≥75	4,083 (31.5%)		21.3 (19.9, 22.7)	
Gender	Men	6,839 (52.8%)	<0.001	33.8 (32.6, 35.0)	<0.001
	Women	6,111 (47.2%)		32.1 (30.9, 33.4)	
Stage	Local	3,849 (29.7%)	<0.001	47.7 (46.0, 49.4)	<0.001
	Regional	5,383 (41.6%)		38.6 (37.2, 40.0)	
	Distant	3,718 (28.7%)		8.07 (7.13, 9.12)	
Year of diagnosis	2003	321 (2.48%)	<0.001	31.2 (26.2, 37.2)	<0.001
	2004	372 (2.87%)		26.8 (22.1, 32.6)	
	2005	423 (3.27%)		26.6 (22.4, 31.5)	
	2006	547 (4.22%)		30.0 (26.0, 34.7)	
	2007	567 (4.38%)		28.3 (24.5, 32.6)	
	2008	628 (4.85%)		29.2 (25.6, 33.2)	
	2009	701 (5.41%)		30.1 (26.6, 34.1)	
	2010	720 (5.56%)		33.6 (30.1, 37.6)	
	2011	791 (6.11%)		33.8 (30.4, 37.6)	
	2012	822 (6.35%)		33.7 (30.4, 37.3)	
	2013	844 (6.52%)		33.8 (30.5, 37.5)	
	2014	826 (6.38%)		34.2 (30.8, 38.0)	
	2015	821 (6.34%)		35.9 (32.6, 39.6)	
	2016	890 (6.87%)		31.9 (28.7, 35.4)	
	2017	936 (7.23%)		31.8 (28.7, 35.3)	
	2018	951 (7.34%)		32.7 (29.6, 36.2)	
	2020	888 (6.86%)		45.9 (42.5, 49.6)	
Deprivation index	1–19% (least)	2,680 (20.7%)	0.065	36.3 (34.4, 38.3)	<0.001
	20–39%	2,504 (19.3%)		35.5 (33.5, 37.6)	
	40–59%	2,657 (20.5%)		32.8 (30.9, 34.8)	
	60–79%	2,569 (19.8%)		29.9 (28.1, 32.0)	
	80–100% (most)	2,540 (19.6%)		30.1 (28.2, 32.2)	

Median survival time of cancer: 42 weeks.

Baseline characteristics and survival probabilities of patients with CCA (*N* = 12,950). Survival was estimated using the Kaplan–Meier method and compared using the log-rank test. Differences in categorical variables, including year of diagnosis, were assessed using the chi-square test.

Survival probabilities varied significantly by age. Patients aged 0–44 years (*n* = 231; 1.8%) had the highest survival probability (46.5%; *p* < 0.001). Survival rates declined progressively with age: 41.3% in patients aged 45–64 years (*n* = 4,060; 31.4%), 34.9% in those aged 65–74 years (35.3%; *n* = 4,576), and 21.3% in those aged ≥75 years (31.5%; *n* = 4,083). This pattern demonstrates a clear age-related decline in survival rates.

In unadjusted analyses, survival probability was slightly higher in males than in females (33.8% vs. 32.1%). Survival rates by disease stage revealed that the localized group had the highest survival probability (47.7%), followed by the regional (38.6%) and distant stages (8.07%), with significant differences observed between the groups. Stratified by year, survival rates were lowest in 2005 (26.6%) and highest in 2020 (45.9%). Higher deprivation levels were associated with lower survival rates, with 36.3% in the least deprived group and 30.1% in the most deprived group. The overall median survival time for all patients with CCA was 42 weeks.

Table 2 summarizes the fixed and random parameters of the multilevel survival models. The random effects in each model were primarily assessed using two key indicators: *σ* (sigma) and the median hazard ratio (MHR). These indicators play an important role in evaluating regional variability across models (20). The MHR values indicated the presence of between-region heterogeneity, with values consistently above 1 suggesting meaningful regional variation. The inclusion of both individual- and area-level variables in Model 2 slightly reduced regional heterogeneity compared with models including only individual-level variables, although substantial variation persisted.

**Table 2:** Parameter estimates from frailty survival model (HR, 95% CI).

Fixed effect		Model 0	Model 1	Model 2
		HR (95% CI)	HR (95% CI)	HR (95% CI)
Individual level				
Age group (years)	0–44		(Ref.)	(Ref.)
	45–64		1.222 (1.007–1.482)^*^	1.214 (1.000–1.473)*
	65–74		1.616 (1.333–1.960)^***^	1.601 (1.321–1.942)***
	≥75		2.713 (2.236–3.292)^***^	2.683 (2.211–3.255)***
Gender	Men		(Ref.)	(Ref.)
	Women		0.967 (0.925–1.011)	0.967 (0.924–1.011)
Stage	Local		(Ref.)	(Ref.)
	Regional		1.401 (1.323–1.483)^***^	1.405 (1.327–1.488)***
	Distant		3.774 (3.553–4.007)^***^	3.774 (3.554–4.008)***
Year of diagnosis	2003		1.240 (1.056–1.456)^**^	1.240 (1.056–1.456)**
	2004		1.399 (1.197–1.635)^***^	1.392 (1.191–1.626)***
	2005		1.383 (1.197–1.598)^***^	1.383 (1.197–1.598)***
	2006		1.307 (1.137–1.503)^***^	1.305 (1.135–1.501)***
	2007		1.218 (1.065–1.392)^**^	1.217 (1.065–1.390)**
	2008		1.324 (1.163–1.507)^***^	1.324 (1.163–1.508)***
	2009		1.250 (1.100–1.420)^***^	1.246 (1.096–1.416)***
	2010		1.185 (1.044–1.346)^**^	1.185 (1.043–1.345)**
	2011		1.045 (0.923–1.183)	1.044 (0.922–1.181)
	2012		1.081 (0.957–1.221)	1.078 (0.954–1.218)
	2013		1.055 (0.933–1.193)	1.054 (0.932–1.192)
	2014		1.049 (0.926–1.187)	1.049 (0.927–1.188)
	2015		(Ref.)	(Ref.)
	2016		1.052 (0.932–1.186)	1.052 (0.933–1.187)
	2017		1.011 (0.897–1.140)	1.015 (0.900–1.144)
	2018		0.981 (0.870–1.106)	0.986 (0.874–1.111)
	2019		0.878 (0.778–0.992)^*^	0.881 (0.781–0.995)*
	2020		0.761 (0.667–0.867)^***^	0.764 (0.670–0.871)***
Area level				
Deprivation				1.057 (1.031–1.083)***
Random part				
σ (95% CI)		0.075 (0.044–0.125)	0.086 (0.056–0.132)	0.075 (0.045–0.123)
MHR		1.074 (1.043–1.127)	1.086 (1.055–1.135)	1.074 (1.044–1.124)

Results of multilevel frailty Cox proportional hazards models. HRs represent adjusted hazard ratios with 95% confidence intervals. *p < 0.05, **p < 0.01, ***p < 0.001. CI, confidence interval; MHR, median hazard ratio; HR, hazard ratio.

Model 0 served as the baseline model, excluding individual-level and deprivation (area-level) variables. The *σ* value was estimated at 0.075 (95% CI: 0.044–0.125), and the MHR was 1.074 (95% CI: 1.043–1.127), suggesting that unique regional variability exists and that regional differences significantly impact risk. Model 1 describes the regional variability, including individual-level variables. The σ value increased slightly to 0.086 (95% CI: 0.056–0.132), indicating that regional variability persisted even after accounting for individual-level factors. The MHR also increased slightly to 1.085 (95% CI: 1.055–1.135). Model 2 provides a more detailed description of regional variability by including the DI as a regional-level variable alongside individual-level variables. Each unit increase in DI was associated with a 5.7% higher risk of mortality (HR 1.057), highlighting the gradient effect of socioeconomic disadvantage. The *σ* value decreased slightly to 0.075 (95% CI: 0.045–0.123), suggesting that deprivation partially explained regional variability. The MHR (1.074, 95% CI: 1.041–1.124) remained similar to that of Model 0.

Comparisons of the random effects across the three models revealed slight shifts in the explanatory power of regional variability with the inclusion of individual-level variables and deprivation. While σ values remained relatively stable between Models 0 and 2, the inclusion of deprivation marginally reduced the MHR, highlighting its significance in explaining regional variability. These findings indicate that deprivation is a key contributor to regional variability and that risk assessment is more accurate when deprivation is considered alongside individual-level factors. Therefore, Model 2 provides a more comprehensive explanation of regional variability, emphasizing the importance of incorporating deprivation in survival analysis.

Figures 1 and 2 present the empirical Bayes estimates derived from Model 2. Figure 1 illustrates the estimated marginal mean differences for significant factors, including (a) year of diagnosis, (b) age group, (c) sex, and (d) stage at diagnosis. Survival rates were lower during the years 2003–2010 but increased after 2015, reaching their highest level in 2020. Survival rates were lower for older age groups and distant-stage diagnoses. In adjusted marginal estimates, females showed slightly higher survival than males; however, this difference was not statistically significant (HR 0.967, 95% CI: 0.924–1.011). This apparent reversal compared with the unadjusted results reflects model-based adjustment rather than a true survival advantage. Figure 2 illustrates the impact of the stage at diagnosis and deprivation level on 1-, 3-, and 5-year survival rates. Reflecting the steeper early decline in survival and attenuation of relative differences over time, the slopes for regional deprivation indices decreased for 3- and 5-year survival rates compared with 1-year survival.

## Discussion

This study demonstrates that both individual and contextual factors significantly influence CCA survival in Busan. Consistent with prior research findings, age, sex, and cancer stage emerged as critical determinants, while the area-level DI independently predicted poorer outcomes.

Patients residing in the most deprived neighborhoods experienced markedly lower survival rates than those in the least deprived areas. Each unit increase in DI was associated with a 5.7% higher risk of mortality (HR 1.057), highlighting the gradient effect of socioeconomic disadvantage. Notably, the inclusion of deprivation in multilevel survival models partially explained regional heterogeneity, emphasizing its explanatory power in survival disparities. However, a substantial proportion of regional variability remained unexplained, suggesting that additional contextual factors, such as healthcare infrastructure, regional referral systems, and availability of specialized care, may also contribute to survival differences. Therefore, deprivation should be interpreted as one of several contributing factors rather than a sole determinant of regional survival differences. These findings align with those of international studies showing that socioeconomic disadvantage contributes to delayed diagnosis, reduced treatment access, and limited supportive care, all of which may worsen cancer outcomes (21–23).

Survival declined sharply with age, with patients aged ≥75 years showing the lowest survival, reflecting the vulnerability of older populations owing to physiological decline, comorbidities, and limited treatment options (24–26). The adverse impact of age was compounded by deprivation, suggesting that older patients in socioeconomically disadvantaged neighborhoods represent the highest-risk group. However, this relationship was not formally tested using interaction terms in the present analysis. Therefore, this finding should be interpreted cautiously, and further studies are needed to evaluate potential effect modification between age and socioeconomic deprivation. This interaction is particularly relevant in Busan, where the rapid transition toward a super-aged society amplifies the burden of deprivation on cancer outcomes. This observation underscores the importance of considering demographic shifts when interpreting cancer survival trends and planning healthcare interventions. In unadjusted analyses, males showed slightly higher survival than females; however, this difference was not statistically significant after adjustment (HR 0.967). In adjusted marginal estimates, females appeared to have slightly higher survival; however, this reflects the modeling approach rather than a true survival advantage and is inconsistent with the unadjusted Kaplan–Meier estimates. Although biological variation may partly explain this gap, disparities in health behaviors, access to care, and social roles could also contribute, raising the need for sex-sensitive approaches to cancer control (27). These findings highlight the need for further investigation into sex-related survival differences, including potential biological and healthcare access factors. Cancer stage remained the strongest predictor of outcome, with survival highest in the localized stage and considerably lower in the distant stage. Notably, the persistence of lower survival rates in highly deprived neighborhoods, even after accounting for cancer stage, suggests that deprivation may delay diagnosis and limit access to optimal treatment (28–30). Taken together, these findings highlight how deprivation contributes alongside individual-level vulnerabilities—older age, and advanced stage—to survival disparities in CCA. In rapidly aging urban settings such as Busan, addressing socioeconomic inequalities is essential. Region-specific strategies that integrate early detection, equitable treatment access, and tailored supportive care for vulnerable populations are critical to improving outcomes and reducing disparities in CCA survival.

Furthermore, although survival improved over time, particularly after 2015, disparities persisted across deprivation levels. The persistently low survival rates among residents in deprived neighborhoods indicate that advances in medical technology and cancer treatment are not equally distributed. This highlights a critical gap in health equity and suggests the need for region-specific interventions to ensure that progress in cancer care translates into improved outcomes for all populations.

These findings are expected to serve as valuable evidence for developing local public health policies, advancing efforts to strengthen health equity, and optimizing treatment outcomes for patients with cancer. Effective cancer management in Busan requires a multifaceted approach that addresses not only early detection and optimized treatment but also the underlying social determinants of health. Interventions such as improving access to specialized cancer centers, expanding welfare and supportive care programs for older adults and socioeconomically vulnerable populations, and tailoring community-based outreach programs can mitigate the negative impact of deprivation on survival. From a health system perspective, addressing disparities in referral pathways to hepatobiliary centers, availability of multidisciplinary cancer care, and regional differences in access to surgical and systemic therapies will be critical. Strengthening coordinated cancer care networks and reducing geographic and socioeconomic barriers to specialized treatment may help translate epidemiologic insights into improved survival outcomes.

This study has a few limitations. First, the use of retrospective registry data may have introduced incomplete clinical information, as details on treatment, comorbidities, and performance status were unavailable. These factors are well-established prognostic determinants in Cholangiocarcinoma and may act as mediators between socioeconomic deprivation and survival. Therefore, the observed association should be interpreted with caution, as it may partially reflect inequalities in treatment access and underlying health status. Second, DI was derived from 2015 census data and applied across the study period, which may not fully capture temporal shifts in socioeconomic conditions. Although commonly used in population-based research, this approach may introduce contextual misclassification. However, socioeconomic deprivation in urban settings tends to reflect relatively stable structural inequalities over time, suggesting that the DI may still capture persistent contextual disparities rather than short-term fluctuations. Future studies incorporating time-varying deprivation measures or period-stratified analyses are warranted. Third, only area-level deprivation was assessed, raising the possibility of ecological bias owing to the absence of individual-level socioeconomic data. Finally, because the study was restricted to an urban, rapidly aging population in Busan, the findings may not be generalizable to other demographic settings.

## Conclusion

This study demonstrated that both individual-level risk factors and area-level deprivation significantly influence survival outcomes in patients with CCA. In a rapidly aging city, such as Busan, the compounded effect of advanced age and socioeconomic disadvantage warrants urgent attention. Public health strategies that integrate demographic realities with social determinants of health are essential to reducing inequalities and improving the prognosis of this highly lethal cancer. Future studies should build on these findings by integrating individual-level socioeconomic factors, comorbidities, and treatment-related data to enhance understanding of survival variations. Moreover, longitudinal research that explores changing patterns of deprivation and evaluates tailored regional strategies may provide opportunities to improve equity and outcomes in Cholangiocarcinoma within rapidly aging urban populations.

## Data Availability

The data analyzed in this study is subject to the following licenses/restrictions: the data are restricted due to ethical and legal considerations related to patient privacy and require institutional approval for access. Requests to access these datasets should be directed to Dong Uk Kim, amlm3@hanmail.net.
